# Visual Analogue Scale for the Evaluation of Olfactory and Gustatory Dysfunction of COVID-19 Patients in Northwestern Greece

**DOI:** 10.7759/cureus.36413

**Published:** 2023-03-20

**Authors:** Athina Zarachi, Aikaterini D Lianou, Vasileios Pezoulas, Ioannis Komnos, Orestis Milionis, Dimitrios Fotiadis, Haralampos Milionis, Ioannis G Kastanioudakis, Angelos Liontos

**Affiliations:** 1 Department of Otorhinolaryngology, Head and Neck Surgery, School of Health Sciences, University Hospital of Ioannina, University of Ioannina, Ioannina, GRC; 2 Department of Otolaryngology, Head and Neck Surgery, General Hospital of Filiates, Ioannina, GRC; 3 Unit of Medical Technology and Intelligent Information Systems, Department of Materials Science and Engineering, Panepistimioupoli Ioanninon, University of Ioannina, Ioannina, GRC; 4 Department of Internal Medicine, School of Health Sciences, University Hospital of Ioannina, University of Ioannina, Ioannina, GRC

**Keywords:** taste, olfaction, visual analogue scale, chemosensory dysfunction, covid-19

## Abstract

Background

The visual analogue scale (VAS) has been used as a diagnostic tool for the evaluation of the severity of olfactory and gustatory dysfunction (OGD) caused by SARS-CoV2 infection. The main objective of the present study was the evaluation of OGD with VAS in COVID-19-positive patients in Northwestern Greece and its possible association with the patients' self-reported symptoms of olfactory and gustatory dysfunction.

Methods

The presence of olfactory and gustatory symptoms and their severity were assessed by questionnaire along with the use of specific odorants and tastant ingredients, in three time periods: prior to COVID-19, during COVID-19 (initial diagnosis) and post-COVID-19 disease (at four weeks from disease onset). Three hundred COVID-19-positive patients (home-quarantined and hospitalized) tested with RT-PCR test in the University Hospital of Ioannina Greece were included in this study. Statistical analysis was performed on SPSS Statistics 26.0 (IBM Corp., Armonk, NY)

Results

Out of a total of 300 patients, 146 and 190 patients had mild hyposmia and hypogeusia respectively, followed by patients with severe hyposmia or hypogeusia (118 and 88 respectively), at the time of COVID-19 onset (initial diagnosis). An increase in the number of patients with recovery of symptoms was observed during the follow-up period, during which only eight patients had non-resolving severe symptoms (six patients with hyposmia and two with hypogeusia).

On further analysis, a statistically significant association was found between the severity of symptoms (assessed by VAS score) and the self-reported symptoms of sensory dysfunction by the patients. There was a significant association between the groups of patients with mild hyposmia and patients that reported no loss of smell; between the patients with moderate hyposmia and the patients who reported "loss of smell"; and between the patients with severe hyposmia and the group of patients who reported a loss of smell, at the COVID-19 onset period. Similarly, patients with mild hyposmia were associated with those that reported a loss of smell at the same time. The severity of hyposmia was also associated with the reported symptom of "loss of taste" at the time of COVID-19 diagnosis. Similar findings were observed regarding the severity of hypogeusia and the reported symptom of “loss of taste” among the groups of patients. Finally, the severity of hypogeusia was associated with smell loss at the time of initial diagnosis of the infection.

Conclusion

Similar to the literature data, our findings indicate that hyposmia and hypogeusia are common symptoms of COVID-19 disease with varying severity. In our study, most of the patients exerted a complete recovery of these OGD symptoms. In addition, we found an association between olfactory dysfunction and self-reported sensory of taste as well as gustatory dysfunction and sensory of smell. Finally, we found that the VAS score was a reliable diagnostic tool in the estimation of OGD in this cohort of patients. However, our results need to be confirmed by larger-scale trials.

## Introduction

Introduction

Previous studies have shown that olfactory and gustatory dysfunction, reported as anosmia and ageusia, are common symptoms in patients with COVID-19 disease [1-7].

The risk factors associated with hyposmia and hypogeusia have been previously studied. A multi-ancestry genome-wide association study by Shelton et al. (n = 69,841) regarding COVID-19-related hyposmia and hypogeusia identified UGT2A1 and UGT2A2 genes expressed in the olfactory epithelium that participate in metabolizing odorants. These genes have been shown to be implicated in the underlying biological mechanisms of COVID-19-associated hyposmia and hypogeusia [8]. In a study by Esposito et al., the neural connectivity of the central olfactory system in patients with persisting hyposmia after SARS-CoV2 infection has been analyzed. It was found that regions functionally connected with the anterior piriform cortex were associated with greater residual olfactory impairment [9]. In addition, in a study by Neta et al., it has been hypothesized that the main cause of olfactory and gustatory disorders is due to the decrease of sensory neurons sensitivity and the co-expression of ACE2 and TMPRSS2 in the epithelial cells [10]. Last, another hypothesis regarding the pathophysiology of olfactory loss is the failure in the activation of sensory neurons in the olfactory epithelium caused by the obstruction of the olfactory clefts [11]. Finally, the underlying mechanism of hypogeusia in COVID-19 has been attributed to the occupation of sialic acid receptors by SARS-CoV2 [12].

It has been shown that the frequency of sensory disorders in COVID-19-affected patients ranged from 5% to 98% [1]. A cross-sectional study on 355 patients from an ENT clinic in Italy showed that the overall prevalence of sensory disorders in COVID-19 patients was 70% [2]. Similarly, in a multicenter European study from 12 hospitals (n = 417) the percentage of COVID-19 patients with OGD ranged from 85.6% to 88% [3]. In addition, in a study by Rojas-Lechuga et al. with 304 patients, it was observed that the frequencies of olfactory and gustatory losses were significantly higher among 197 COVID-19 patients (70.1% and 65.0%, respectively) [4]. Another case-control study with 320 patients also reported on the incidence of OGD in health workers; the incidence of OGD was 64.1% for olfactory and 60.5% for gustatory dysfunction, respectively [5]. Finally, in a retrospective study (n = 114) by Klopfenstein et al., it was found that 47% of COVID-19 patients presented with anosmia [7]. On the other hand, results from a small-sized study (n = 74) showed a lower incidence of OGD [6]. In this study, 31.1% and 44.6% of patients presented with olfactory and gustatory disorders, respectively [6].

Visual analogue scale (VAS) was used as a diagnostic tool for the evaluation of olfactory and gustatory dysfunction (OGD), in previous studies [4,6,13,14] and European Position Paper on Rhinosinusitis and Nasal Polyps (EPOS) guidelines proposed a classification for sinonasal symptoms, using VAS. According to this scale, all related symptoms can be classified as mild (VAS: 0-3), moderate (VAS > 3-7) and severe (VAS > 7-10) disease [15].

Izquierdo-Domínguez et al. proposed the use of a questionnaire that included (a) a smell loss VAS (score scale 0-10; 0: no loss of smell and 10: maximum loss of smell) and (b) a taste loss VAS with the same scoring range [13]. Based on EPOS guidelines [15], investigators classified patients as normosmic-mild (VAS 0-3), moderate (VAS 4-6) and severe olfactory loss (VAS 7-10) [13]. In the study by Rojas-Lechuga et al., the assessment of olfactory/gustatory function was performed with a questionnaire including VAS (score scale 0-10; 0: no loss of smell/taste and 10: maximum loss of smell/taste) [4]. However, compared to the study by Izquierdo-Domínguez et al., [13] investigators in this study did not use EPOS guidelines, due to limitations related to the severity of the COVID-19 pandemic. In another single-centre study of COVID-19 patients in Japan, olfactory and gustatory disorders were assessed using a reversed VAS score (0: worst sensory function and 100: excellent sensory function) [6]. Similarly, in the study by Iannuzzi et al., investigators requested the patients to quantify their olfactory function, also using the VAS score system. According to this, the severity of symptoms was categorized as follows; 0: totally impaired olfaction and 100: an excellent sense of smell [14].

The aim of the present study was to assess the severity of olfactory and gustatory symptoms in a cohort of COVID-19-positive patients from Epirus, Greece, with the use of a VAS score. Alteration and recovery of symptoms during the course of the disease and at the follow-up time were also examined. In addition, the VAS scoring classification of patients and its possible association with the self-reported symptoms of OGD was also examined.

## Materials and methods

Materials and methods

Participants

This was a prospective observational study. A total of 300 patients (randomly selected) aged from 16 to 90 years old, were included in this study. All patients were referred to the Emergency Department (ED) or to the outpatient clinic of Infectious Diseases at the University General Hospital of Ioannina (UGHI). According to disease severity, the studied population included both home-quarantined patients with mild to moderate disease and patients with severe disease that were hospitalized in the Infectious Diseases Unit (IDU) of the UGHI.

In order to participate in the study, each patient required a positive RT-PCR test for SARS-CoV-2. The recruitment period of this study was from November 2020 to May 2021. The inclusion criteria of the study were patients with the following characteristics: adults ≥ 16 years old and ≤ 90 years old and a positive RT-PCR COVID-19 test performed at the UGHI. The exclusion criteria of the study concerned patients with a history of rhinosurgery, endoscopic sinus surgery or radiotherapy of the head and neck. Patients with rhinitis (allergic or not), chronic rhino sinusitis, or a history of head trauma were also excluded from this study.

Data Collection

Data collection for this study was performed with the use of a questionnaire. Patients’ demographics, reported symptoms and the results of the clinical examination were documented in the questionnaire. Patients were requested to evaluate olfactory and gustatory function in three time periods: prior to COVID-19, during COVID-19 (initial diagnosis) and post-COVID-19 disease (at four weeks from disease onset). All questions in the study questionnaire were used for the evaluation of OGD severity. Answers were documented per patient and per time-point.

The initial examination and evaluation of olfactory and gustatory function were performed at the onset of COVID-19 disease; time of diagnosis with RT-PCR or a few days later (up to three days from initial diagnosis). Patients were requested to recall olfactory and gustatory functions prior to the infection. All patients underwent a follow-up examination four weeks after the diagnosis.

The data collection procedure differed for each group of patients. Home-quarantined patients were informed of the study and their enrollment by telephone, e-mail or in person following all safety measures according to the National Organization of Public Health of Greece guidelines. Hospitalized patients in the IDU of the UGHI were examined in person. Investigators provided a written consent form as well as detailed information about the aim of the study to every participant. All participants provided a signed consent form prior to their enrollment. Evaluation of patients was done by completion of a specific questionnaire as indicated by the study protocol.

The questionnaire used in this study included: (a) a visual analogue scale (VAS) score for the evaluation of loss of olfactory function (VAS score: 0-10; 0: a normal sense of smell; 10: total anosmia); (b) a VAS for the evaluation of loss of gustatory function (VAS score: 0-10; 0: a normal sense of taste; 10: total ageusia). The questionnaire also included: (c) the same VAS scale categorization for specific odorants (lemon, oregano, instant coffee, toothpaste, mint chewing gum), and (d): the same VAS scale categorization for specific tastes (sugar, salt, lemon, instant coffee). The rationale for the use of these odorants is their ability to stimulate the olfactory nerve and both olfactory and trigeminal nerve (unimodal and bimodal odours) [16,17]. Similarly, these tastants were used to emphasize the taste perception of salty, sweet, bitter, and sour/acidic [1,16]. All patients were requested to evaluate olfactory and gustatory intensity in the aforementioned time periods: prior to COVID-19 disease, during periods of COVID-19 and post-COVID-19 infection four weeks from disease onset.

According to EPOS guidelines classification of OGD with VAS [18], we categorized our patients into specific groups. In our study, VAS in COVID-19 patients categorized the quantitative variables of hyposmia/ hypogeusia into three groups based on the severity of OGD. Thus, analyzing the results from the questionnaire, we defined mild hyposmia and hypogeusia according to the sensory function in patients with a VAS score of 0-3, moderate hyposmia/hypogeusia in patients with a VAS score of 4-7, and severe hyposmia/hypogeusia in patients with VAS score of 8-10. Of note, this categorization was performed considering that patients’ answers were given on a self-estimate of sensory function. All median VAS scores for all odorants and tastes were calculated, in the three time periods.

Ethical approval was granted by the Institutional Ethics Committee of the University General Hospital of Ioannina with Protocol Number: 1/6-4-2021 (issue:257) and ClinicalTrials.gov Identifier: NCT04657445.

Statistical analysis

The statistical analysis regarding categorical data was performed using the x2 (chi-square) test or Fisher’s exact test, where at least one frequency in the contingency table was < 5. 

Percentages of patients of each of the three groups with VAS score (0-3, mild hyposmia/hypogeusia; 4-7, hyposmia/hypogeusia; 8-10, severe hyposmia/hypogeusia), were calculated in the entire study population. Further analysis of these results with the self-reported symptoms "loss of smell" and "loss of taste" during the infection, were also performed.

## Results

Results of the analysis of the entire cohort (n = 300 patients) were as follows; 299 patients (99.67%) reported mild hyposmia (VAS score: 0-3) prior to COVID-19 disease time period, while a single patient (0.33%) reported moderate hyposmia (VAS score: 4-7). None of the patients had severe hyposmia (VAS score: 8-10) prior to the infection. Of note, all patients (n = 299) that reported mild symptoms were considered normosmic as they had a VAS score of 0 or 1. A total of 146 patients (48.66%) reported mild hyposmia during COVID-19 disease (at the time of initial diagnosis) while 36 patients (12.0%) and 118 patients (39.33%) reported moderate and severe hyposmia during this period, respectively. At the time of the follow-up period (post-COVID-19 disease at four weeks from disease onset), 285 patients (95.0%) reported mild hyposmia in the entire study cohort. Nine patients (3.0%) reported moderate hyposmia and six patients (2.0%) reported severe hyposmia (Table 1).

**Table 1:** Number and percentages of patients with mild, moderate and severe hyposmia at three time periods: before, at the time of diagnosis and four weeks at the follow-up, in the entire cohort. Mild hyposmia patients' VAS score: 0-3; moderate hyposmia patients' VAS score: 4-7; severe hyposmia patients' VAS score: 8-10.

Feature	Entire cohort of patients (n=300)	Percentage of patients in the entire cohort (n=300) %
Time-period/symptoms		
Period: prior to COVID-19 disease		
Mild hyposmia	299	99.66
Moderate hyposmia	1	0.33
Severe hyposmia	0	0.00
Period: during COVID-19 disease (at initial diagnosis)		
Mild hyposmia	146	48.66
Moderate hyposmia	36	12.00
Severe hyposmia	118	39.33
Period: post-COVID-19 disease (at 4 weeks from disease onset)		
Mild hyposmia	285	95.00
Moderate hyposmia	9	3.00
Severe hyposmia	6	2.00

Regarding the symptom of hypogeusia in the entire cohort, all patients (n = 300, 100%) reported mild hypogeusia prior to the COVID-19 disease duration. No patient (0.0%) reported moderate or severe hypogeusia. During the infection period, 190 patients (63.33%) reported mild hypogeusia, 22 patients (7.3%) reported moderate hypogeusia and 88 patients (29.33%) reported severe hypogeusia. At the time of follow-up, 295 patients (98.33%) reported mild hypogeusia, 3 patients (1.0%) reported moderate hypogeusia and two patients (0.66%) reported severe hypogeusia (Table 2).

**Table 2:** Number and percentages of patients with mild, moderate and severe hypogeusia at three time periods: before, at the time of diagnosis and four weeks at the follow-up in the entire cohort. Mild hypogeusia patients' VAS score: 0-3, moderate hypogeusia patients' VAS score: 4-7, severe hypogeusia patients' VAS score: 8-10.

Feature	Entire cohort of patients (n=300)	Percentage of patients in the entire cohort (n=300) %
Time period/symptoms		
Period: prior to COVID-19 disease		
Mild hypogeusia	300	100.00
Moderate hypogeusia	0	0.00
Severe hypogeusia	0	0.00
Period: during COVID-19 disease (at initial diagnosis)		
Mild hypogeusia	190	63.33
Moderate hypogeusia	22	7.33
Severe hypogeusia	88	29.33
Period: post-COVID-19 disease (at 4 weeks from disease onset)		
Mild hypogeusia	295	98.33
Moderate hypogeusia	3	1.00
Severe hypogeusia	2	0.66

Further analysis was performed on the association between documented VAS scores of olfactory and gustatory function and the self-reported symptoms "loss of smell" and "loss of taste". 

A significant association was observed between the group of patients with mild hyposmia were less likely to report "loss of smell" (OR: 0.016, p <0.0001), at the time of COVID-19 diagnosis. On the other hand, patients with moderate hyposmia at the same time period, had greater odds to report "loss of smell" (OR: 7.2, p < 0.0001). This finding was also observed between the group of patients with severe hyposmia and the group of patients that reported "loss of smell", during the initial diagnosis of the infection (OR: 38.9, p < 0.0001).

At the follow-up period (four weeks), patients with mild hyposmia had lower risk of lingering "loss of smell" (OR: 0.19, p = 0.017). Similar to this, a significant association was found between the group of patients with severe hyposmia and the patients that reported "loss of smell" during the post-COVID-19 period (p = 0.039) (Table 3).

**Table 3:** Associations between the groups of patients with mild, moderate and severe hyposmia and the patients with reported symptom "loss of smell", at three time periods: before, at the time of diagnosis and four weeks at the follow-up. Mild hyposmia patients' VAS score: 0-3; moderate hyposmia patients' VAS score: 4-7; severe hyposmia patients' VAS score: 8-10.

Feature	Reported symptom: "loss of smell"	Reported symptom: "No loss of smell"	p-value
Time period/ symptoms			
Period: prior to COVID-19 disease			
Mild hyposmia	171	128	0.43
Moderate hyposmia	0	1	0.43
Severe hyposmia	0	0	1.0
Period: during COVID-19 disease (at initial diagnosis)			
Mild hyposmia	27	119	<0.0001
Moderate hyposmia	32	4	<0.0001
Severe hyposmia	112	6	<0.0001
Period: and post-COVID-19 disease (at 4 weeks from disease onset)			
Mild hyposmia	158	127	0.017
Moderate hyposmia	7	2	0.309
Severe hyposmia	6	0	0.039

Patients with mild hyposmia at the time of infection had a lower risk to report the symptom "loss of taste" (OR: 0.07, p <0.0001). On the other hand, patients with moderate hyposmia had a greater risk to report the symptom "loss of taste" (OR: 3.8, p = 0,002). In addition, a similar risk was found in the group of patients with severe hyposmia and the reported symptom "loss of taste" (OR: 8.4, p < 0.0001), at the time of diagnosis of COVID-19 (Table 4).

**Table 4:** Associations between the groups of patients with mild, moderate and severe hyposmia and the patients with reported symptom "loss of taste" at three time-periods: before, at the time of diagnosis and four weeks at the follow-up. Mild hyposmia patients' VAS score: 0-3; moderate hyposmia patients' VAS score: 4-7; severe hyposmia patients' VAS score: 8-10

Feature	Reported symptom: "loss of taste"	Reported symptom: "No loss of taste"	p-value
Time period/symptoms			
Period: prior to COVID-19 disease			
Mild hyposmia	155	144	0.483
Moderate hyposmia	0	1	0.483
Severe hyposmia	0	0	1.0
Period: during COVID-19 disease (at initial diagnosis)			
Mild hyposmia	32	114	<0.0001
Moderate hyposmia	28	8	0.002
Severe hyposmia	95	23	<0.0001
Period: and post-COVID-19 disease (at 4 weeks from disease onset)			
Mild hyposmia	146	139	0.691
Moderate hyposmia	5	4	1.0
Severe hyposmia	4	3	0.685

In the analysis between the groups of patients with hypogeusia and patients that reported "loss of taste" it was observed that the odds of self-reported "loss of taste" at the time of initial diagnosis were lower in patients with mild hypogeusia and significantly higher in patients with moderate hypogeusia (OR: 0.01 and 22.6, respectively, all p < 0.0001). In the group of patients with severe hypogeusia risk of reported "loss of taste", at the same time period was also significantly high (OR: 57.5, p <0.0001), (Table 5).

**Table 5:** Associations between the groups of patients with mild, moderate and severe hypogeusia, before, during and after infection and the group of patients with reported symptoms "loss of taste", at three time periods: before, at the time of diagnosis and four weeks at the follow-up. Mild hypogeusia patients' VAS score: 0-3; moderate hypogeusia patients' VAS score: 4-7; severe hypogeusia patients' VAS score: 8-10

Feature	Reported symptom: "loss of taste"	Reported symptom: "No loss of taste"	p-value
Time period/ symptoms			
Period: prior to COVID-19 disease			
Mild hypogeusia	155	145	1.0
Moderate hypogeusia	0	0	1.0
Severe hypogeusia	0	0	1.0
Period: during COVID-19 disease (at initial diagnosis)			
Mild hypogeusia	49	114	<0.0001
Moderate hypogeusia	21	1	<0.0001
Severe hypogeusia	85	3	<0.0001
Period: and post-COVID-19 disease (at 4 weeks from disease onset)			
Mild hypogeusia	150	145	0.061
Moderate hypogeusia	3	0	0.248
Severe hypogeusia	2	0	0.499

A lower risk of self-reported "loss of smell" was found in the group of patients with mild hypogeusia (OR: 0.06, p < 0.0001), at the time of COVID-19 diagnosis. On the contrary, a higher risk was observed in the group of patients with moderate and severe hypogeusia during this time period (OR: 5.25, p= 0.003 and OR: 15.7, p < 0.0001) (Table 6).

**Table 6:** Associations between the groups of patients with mild, moderate and severe hypogeusia and the patients with reported symptom "loss of smell", at three time periods: before, at the time of diagnosis and four weeks at the follow-up. Mild hypogeusia patients' VAS score: 0-3; moderate hypogeusia patients' VAS score: 4-7; severe hypogeusia patients' VAS score: 8-10

Feature	Reported symptom: "loss of smell"	Reported symptom: "No loss of smell"	p-value
Time period/ symptoms			
Period: prior to COVID-19 disease			
Mild hypogeusia	171	129	1.0
Moderate hypogeusia	0	0	1.0
Severe hypogeusia	0	0	1.0
Period: during COVID-19 disease (at initial diagnosis)			
Mild hypogeusia	71	119	<0.0001
Moderate hypogeusia	19	3	0.003
Severe hypogeusia	81	7	<0.0001
Period: and post-COVID-19 disease (at 4 weeks from disease onset)			
Mild hypogeusia	166	129	0.073
Moderate hypogeusia	3	0	0.262
Severe hypogeusia	2	0	0.508

## Discussion

Our results provide new insights into the clinical course of OGD and the relationship between these disorders in COVID-19 patients. Numerous researchers have investigated the effect of SARS-CoV-2 on the olfactory and gustatory functions of infected patients [1-7, 13].

The incidence of olfactory and gustatory disorders is estimated to be approximately 50% of patients (47.85% and 47.5%, respectively) worldwide [19,20].

In the systematic review by Jafar et al. [21], investigators assessed data from 14 studies which included patients with mild to moderate symptomatology. Results of the analysis showed that in studies with objective evaluation tests, less than 40% of patients reported normosmia at initial evaluation. On the contrary, in studies with subjective testing from patients, normosmia could range from 0% to 90% at initial evaluation [21]. Our study was a questionnaire-based survey on OGD in home-quarantined and hospitalized patients with COVID-19 referred to our hospital. Similarly to literature data, in our study, approximately half or greater than half of patients (44.66% and 63.33%) reported mild hyposmia and hypogeusia, respectively, at the time of COVID-19 onset (initial diagnosis). On the other hand, the incidence of severe symptoms was lower (39.33% and 29.33%, respectively).

Regarding the recovery rate of symptoms in our study, it was observed an increase in the number of patients with full or partial recovery during the follow-up period (at four weeks); only six and two patients (2.00% and 0.66%, respectively) had non-resolving severe symptoms (hyposmia and hypogeusia, respectively) from the entire cohort. Similar findings were reported in the study by Koul et al. [22]. From the 300 patients enrolled, 50% reported single OGD (olfactory: 55.33% and gustatory: 15.33%), while 46 patients (30.66%) reported combined OGD. The follow-up examination was four weeks after the initial diagnosis [22]. Most of these patients reported a complete or partial recovery of olfactory function (78% and 18%, respectively). A total of six patients (4%) had no improvement in olfactory function, while all patients with gustatory symptoms reported complete improvement [22]. The evaluation of patient symptoms in this study was performed using a standardized questionnaire proforma. Similarly to this, in our study, we also used a questionnaire to evaluate OGD.

On further analysis of our results, we found significant associations between the severity of symptoms (assessed by VAS score) and the self-reported symptoms by the patients, of sensory dysfunction. As mentioned before VAS has been used to assess olfactory and gustatory dysfunction (OGD), in previous studies [4,6,13-15]. All these studies showed that a higher VAS score was associated with more severe OGD [4,6,13].

In our study we used VAS categorization based on EPOS criteria to evaluate OGD at three time points; before, during (at the first days) and after SARS-CoV2 infection. Our findings showed that all patients (except one) had mild hyposmia (VAS < 3) before the infection. All these patients were considered normosmic. The majority of patients in our study exerted mild hyposmia during the infection (in the first days of the initial diagnosis), followed by patients with severe hyposmia. On the other hand, a minority of patients had moderate hyposmia. Similar results were found from the evaluation of gustatory function. The results of our study regarding VAS categorization in the entire cohort of patients are in line with the findings by Yamamoto et al. [6]. In this study, the mean VAS for olfaction (overall) and gestation (food) was 19 and 31.5, respectively (0: worst sensory function - 100: excellent sensory function) while the sense of smell was particularly impaired. Data analysis showed that the mean VAS score declined (0.5 and 20, respectively), at the time of the most severe symptoms [6].

However, our findings differ from the study by Rojas-Lechuga et al [4]. Investigators have shown that the frequency and severity of OGD in COVID-19 patients were higher compared to controls with common cold-like symptoms [4]. In this study, however, the majority of patients with olfactory dysfunction had severe smell loss (higher VAS score), followed by patients with moderate symptoms, while the minority of patients exerted mild symptoms. Similarly to this, it was shown that the majority of patients with gustatory dysfunction had severe taste loss, while the minority had mild taste loss. It was also shown that patients with mild OGD (low VAS of 0-3) had shorter recovery periods [4].

At the time of the follow-up (four weeks later), most of the patients in our study reported mild hyposmia, while the minority reported moderate and severe hyposmia. The complete recovery of hyposmia was reported by most of the patients in our study. Similar results regarding this finding were also observed in the study by Riesta-Ayora et al. [5]. In this six-month case-control study of health workers, the recovery rate in the case group of patients and in the control group was assessed. Results showed that in both groups, OGD had high-resolution rates during the first two months after the onset of symptoms, while 30% of patients showed partial recovery after six months. A percentage of 11% did not show any recovery. In addition, a significant association was found between olfactory dysfunction and gustatory dysfunction [5].

In our study, a similar association was also observed between the group of patients with mild, moderate or severe hyposmia and the self-reported symptom "loss of smell". A similar association was observed between the group of patients with mild, moderate or severe hypogeusia during the infection and the reported symptom of "loss of taste". Results from a study (n = 162 patients) on the long-term assessment of OGD during COVID-19 disease, showed that the recovery period was higher in the severe anosmia group (VAS ≥ 7) compared to the non-severe group (VAS < 7) [31.9 days vs 22.9 days] [23]. In a systematic review by Jafar et al., olfactory recovery was observed in the first two weeks after the infection while it could extend in patients with persistent anosmia for up to six months. In this analysis, poor olfaction at initial presentation was associated with poor recovery rates [14]. However, in our study, we did not evaluate the time needed for symptom recovery but the percentage of patients at the follow-up. 

Previous studies showed that the incidence of taste loss is similar to smell loss. In this perspective, questions have been raised regarding the difference between loss of flavour (smell and taste) and loss of real taste (sweet, salty, bitter, and sour/acidic) [24]. In the pilot study by Kaye et al., [25], gustatory dysfunction was considered a consequence of olfactory dysfunction. On the contrary, other studies have clearly differentiated loss of smell and taste [13, 26]. Song et al., have shown that loss of smell is less frequent than loss of taste in hospitalized patients, while loss of taste is associated with severe disease [26].

In our study, it was also shown that hyposmia was associated with the reported symptom of “loss of taste” at the time of COVID-19 diagnosis and hypogeusia was associated with the reported symptom of smell loss, at the same time. In this perspective, we found that the severity of OGD assessed by VAS in our study was associated with a higher risk of self-reporting olfactory and gustatory loss. This finding is indicative of the aforementioned overlap between olfactory and gustatory dysfunction.

Of note, in our study, we excluded all patients with a history of rhinosurgery or endoscopic sinus surgery, as well as patients that underwent radiotherapy of the head and neck. Patients with rhinitis (allergic or not), chronic rhino sinusitis, or a history of head trauma were also excluded from this study. Based on literature data, it has been shown that all these conditions can potentially alter olfactory and gustatory functions [18,27-37].

Our study has some limitations. First, the small number of patients in the present study could explain the absence of significant differences between all groups of patients with varying disease severity. Second, subjective evaluation tests are not always reliable and can thus increase bias. The use of other tests for olfactory and gustatory function for a more detailed study would be desirable in our research.

## Conclusions

The aim of this study was to access olfactory and gustatory function and concomitant disorders at specific time points of COVID-19 disease (prior infection, at initial diagnosis and early stages, and after the infection) using the VAS. To our knowledge, this is the first study that has been conducted in Northwestern Greece regarding the evaluation of olfactory and gustatory disorders during COVID-19 disease that uses VAS score and categorization. In our study, the majority of patients presented with mild hyposmia and mild hypogeusia during SARS-COV2 infection. On the other hand, a minority of patients presented with moderate hyposmia and moderate hypogeusia during the infection. In addition, complete recovery of symptoms indicative of OGD was achieved in the majority of COVID-19 patients as shown in the follow-up period. Our findings dictate that the subjective, self-reported symptoms for OGD during COVID-19 disease are reliable and are associated with the evaluation of OGD using VAS.
